# Association between Depression and Factors Affecting Career Choice among Jordanian Nursing Students

**DOI:** 10.3389/fpubh.2017.00311

**Published:** 2017-11-22

**Authors:** Said Yousef, Mariam Athamneh, Emad Masuadi, Haitham Ahmad, Tom Loney, Hamdy F. Moselhy, Fatma Al-Maskari, Iffat ElBarazi

**Affiliations:** ^1^Department of Psychiatry, College of Medicine and Health Sciences, United Arab Emirates University, Al Ain, United Arab Emirates; ^2^Jordan University of Science and Technology, Irbid, Jordan; ^3^King Saud bin Abdulaziz University, College of Medicine, Riyadh, Saudi Arabia; ^4^College of Medicine and Health Sciences, Institute of Public Health, United Arab Emirates University, Al Ain, United Arab Emirates

**Keywords:** career choice, nursing students, depression, religion, nursing role, Jordan, nursing education

## Abstract

**Background:**

Although stress reaction is high among nursing staff and nursing students in the Middle East and its effect on life is known, there are scant studies reporting on these clinical and social features. In addition, there are no studies reporting on factors that influence career choice among this group.

**Aim:**

This study aimed to investigate factors that influence career choice among nursing students and their possible association with depressive symptoms.

**Method:**

Participants were 150 (84.7% response rate) nursing students randomly selected from each academic year at the Nursing College/Jordan University of Science and Technology. Participants consented and completed the socio-demographic data collection sheet. The Arabic version of the Beck Depression Inventory-II Scale was used to assess participants with respect to depressive symptoms. A modified list of factors influencing career choice and a Likert scale to assess the level of sadness and the degree of religiosity were used as well.

**Results:**

Students ranked the most important three factors influencing their career selection as family decision, religious factors, and the desire to care for others. The prevalence of depression among the sample was 26%. Students who had a desire to care for others were less likely to suffer from depression and those who chose nursing as their career due to religious factors were significantly less depressed than those who did not. Meanwhile, students who chose nursing under family pressure or because of a lack of alternative opportunities were more depressed. The odds ratio for depressive symptoms was 0.24 when students chose nursing because of religious factors, whereas it was 4.92 when the family strongly influenced the student’s career decision and 3.61 when a nursing career was the only perceived opportunity available.

**Conclusion:**

The main factors associated with depression among this sample of nursing students were pressure from their family to choose a nursing career and having no other career or employment opportunities. Religiosity was negatively associated with depression and may act as a protective factor; however, future studies using longitudinal designs will need to confirm this hypothesis.

## Introduction

It is well recognized that nursing professionals experience high levels of stress, anxiety, and depression in comparison to other professions from medical and non-medical fields (1, 2). Stress and anxiety may lead to burnout and to other psychological problems such as anxiety and depression (3, 4). Previous research has identified nursing students as having elevated stress levels compared to college students studying on non-nursing programs (5–7). Earlier work has reported that the main stressors for nurses and nursing students included: adjusting to a rigorous program of theory, long hours of study, and the pressures of student clinical practice (6–11). Prolonged Exposure to stress may lead to numerous stress symptoms and consequences in nursing students who have somatic symptoms, anxiety disorder, depressive symptoms, and cognitive symptoms, which may have a negative impact on academic success (6, 12, 13). Long-term exposure to stressors may lead to anxiety and dissatisfaction at work and possibly depression (14). Furegato and colleagues (15) define depression as a “syndrome characterized by a group of symptoms with changes in mood, behavior, thought, and perception patterns, with physical complaints and a higher risk of suicide.” Moreover, depression can result from overexposure to stress among nursing students (14). Specifically, the symptoms of depression may start manifesting at younger ages during adolescence or early adulthood and is usually more common among women, especially due to hormonal changes during puberty and adolescence (15). This finding raises concerns about the mental well-being of nursing students being more prone to develop depression during their studying years and early career (15). To add to these concerns, previous studies have identified a high prevalence of depression among university students in general and the prevalence has been found to range between 10 and 85%, with a weighted-mean prevalence of 31% (16). An episode of depression prior to college, a family history of treated depression, low self-confidence, self-blame, stress, isolation, a perceived lack of control, and resignation have all been reported as possible risk factors for depression in nursing students (17). Depression is also reported to be associated with unstable personal relationships, suicidal thoughts and attempts, and poorer work performance (16).

A systematic review of depression and anxiety prevalence among medical students found their overall psychological distress to be consistently higher than in the general population (18). Moreover, a high level of distress has been shown to negatively affect academic performance and was associated with higher levels of alcohol and substance abuse among students (17). Finally, distress has been found to be correlated with an unwillingness to care for the chronically ill, decreased empathy, and increased cynicism (18). Globally, numerous studies have identified high levels of stress and depressive symptoms among nursing students. For example, in Sweden, nurses’ depressive symptoms were reported to increase throughout their educational years, after graduation, and at the start of their professional clinical work. However, depression scores have been shown to fall down with experience and a greater number of years working as a nurse suggests that nursing students and early career nurses may be at a higher risk of developing depression (17). In Greece, the prevalence of depressive symptoms among nursing students was reported between 44 and 52% (19, 20). While in Thailand, 50% of nursing students scored above the cutoff point of depression scale (21). In Middle Eastern nursing students, 44% were classified as mildly to moderately depressed, with feelings of hopelessness (22). A systematic review investigating the prevalence of burnout among healthcare professionals in the Arab world reported high levels of burnout among nurses compared to other healthcare professionals suggesting that nurses are at a high risk of developing burnout in this region (23). Another study explored the role of depression on career choice among different professionals including nursing and found that perceived self-concept and identity-related career decision-making difficulties were associated with depressive symptoms for both men and women (24). Previous research has highlighted the importance of career counseling to help students match their skills to ease anxiety and improve their self-efficacy (25–27).

Numerous studies identified a range of different reasons affecting career choices among nursing students and professionals such as the motivating influence of existing nurses acting as role models, the appeal of nurturance and caring for others, employment opportunities, financial benefits, job security, interest in science, and the opportunity to work in a variety of settings (28). Research on factors behind choosing a nursing career is scant in Arab countries and the Middle East region. A study conducted in nursing students from both Turkey and the United States revealed that the main reasons behind their career choices was living conditions and survival needs for Turkish students, whereas American students primarily considered suitability of a profession for themselves (29). Other studies found that altruistic motivation was a common reason for pursuing a nursing career (30, 31).

To our knowledge, there are no previous data on reasons for nursing career choices in the Arab world. Moreover, no studies have been conducted to determine the prevalence of depression among Jordanian students and to explore whether depression is associated with career choices and their perceptions toward their career. Finally, there are no studies that have explored factors affecting career choices among Jordanian students in relation to their cultural and social context.

This study aimed to determine the prevalence of depressive symptoms among nursing students in Jordan and to assess the factors associated with pursuing nursing as a career choice. The main objective of this study was to find out whether career choices among nursing students are associated with depressive symptoms. Another objective was to explore the relationship between religiosity and depression in nursing students.

## Subjects and Methods

### Participants and Procedure

The required sample size was 144 students assuming a depression prevalence of 20%, a precision of 5%, a two-sided 95% confidence interval, and a margin of error of 6.2%. We assumed a non-participation rate of at least ~20% based on previous studies conducted in the Nursing College (unpublished data). This study employed a cross-sectional design using a multistage clustered sampling procedure to recruit a representative sample of nursing students from a large nursing college in Jordan. There was a total population of 344 nursing students enrolled at the Nursing College/Jordan University of Science and Technology (JUST) during the 2012–2013 academic year. The primary goal was to recruit a random representative sample of the total population. All 344 nursing students were assigned a number and then a computerized random number generator was used to select a random proportionate sample of 177 students (~50% of study population) across the four year groups. Selected students were then contacted by email to participate in the study. One-hundred and fifty students (84.7% response rate) agreed to take part in the study and completed the questionnaire in a classroom with students from their year group. A member of the research team supervised completion of the questionnaires in case students had questions related to specific items on the questionnaire. The supervising researcher did not influence the students’ responses. Participants were allowed to withdraw from the study at any point. Participating students completed the questionnaire 1 month prior to the final evaluation exam (October 15, 2012–December 5, 2012) and this time period was chosen to minimize the possible influence of the examination period on stress and depression.

Each subject received information about the nature and the goal of the study. Written informed consent was obtained from all subjects prior to distribution of the questionnaire. This study was reviewed and approved by the Institutional Review Board of King Abdullah University Hospital, JUST/Jordan (No. 3/2012 -48). With regard to quality assurance for data analysis, standard data entry checking procedures, algorithms, and analytical checks were performed. Double data entry was not performed.

#### Measures

A *pro forma* data collection sheet was used to record socio-demographic information (e.g., gender, nationality, smoking status, financial problems, previous health and psychiatric problems). A validated Arabic version (32, 33) of the Beck Depression Inventory-II Scale (BDI185 II) was used to assess the depressive symptoms (Supplementary Material 2). The internal consistency of the Arabic version of the BDI-II has been tested among college students in Jordan (alpha coefficient = 0.86), and in another 17 Arabic countries (alpha coefficient ranged from 0.82 to 0.93) demonstrating good internal consistency (34). This has been validated for use with non-psychiatric patients, including college students (18), and to identify the presence and severity of depressive symptoms consistent with the criteria of the DSM-IV (7). A specially-designed questionnaire based on previous research (28, 35–37) was developed to assess nursing career choices and piloted on 30 student nurses not involved in the main study (Supplementary Material 1). The questionnaire contained a list of 10 factors that had been reported to influence nursing career choice in previous research (27, 28, 35, 36) and participants were required to answer “Yes/No” as to whether this factor was related to their career choice. In addition, participants had the option of including additional factors not considered in the list and were asked to rank the top three most important factors influencing their nursing career choice. Both level of sadness and degree of religious belief were self-reported by respondents through a Likert scale from one (“not at all”) to seven (“very sad”), or at highest level of religious belief.

#### Analysis

Data analysis was performed using the Statistical Package for Social Sciences (SPSS, version 20). Descriptive statistics were calculated for demographic data to show the respondents’ characteristics. A chi-square (χ^2^) test was used to assess the relationship between depression in the BDI-II (cutoff score 17 indicating borderline clinical depression) and categorical data, such as age groups, gender, nationality, birth order, year of study, father and mother educational level, and career choice factors. A Mann–Whitney test was used to examine the differences between the normal and depressed groups in sadness level and degree of religious belief. Multiple logistic regression analysis was used with the BDI-II cutoff score of 17 as the dependent variable and the significant factors affecting their career choice as independent variables. The alpha level was set at ≤0.05.

## Results

### Demographic Data

Demographic characteristics of the participants are summarized in Table 1. Of the 150 student-respondents, only 41 (27%) were male and the majority were 21 years old. Only nine (6%) students were non-Jordanian and approximately one-third were first-born children. The study included all years of the undergraduate nursing students with proportionate samples from each year group. More than half (60%) of participants’ mothers were reported to have secondary school level or below, whereas fathers’ highest education were evenly distributed between secondary school or below.

**Table 1:** Baseline demographic and family characteristics of participants.

Variable	Categories	*N*	%
Gender	Male	41	27.3
	Female	109	72.7

Age	19	19	12.7
	20	37	24.7
	21	74	49.3
	22+	20	13.3

Nationality	Jordanian	141	94.0
	Other nationalities	9	6.0

Birth order	1	38	32.2
	2	18	15.3
	3	19	16.1
	4	13	11.0
	5+	30	25.4

Year of study	Year 1	27	18.0
	Year 2	31	20.7
	Year 3	46	30.7
	Year 4	46	30.7

Father education	Secondary school or below	67	49.6
	Above secondary school	68	50.4

Mother education	Secondary school or below	81	60.0
	Above secondary school	54	40.0

### Depression and Demographic Data

Based on the BDI-II (cutoff score 17), the prevalence of depression among nursing students was 26%. The prevalence of depression among males and females was reported to be 27.3 and 25.5%, respectively, with no significance difference (*P* = 0.837). There was no significant relationship between demographic characteristics and depression status (Table 2). Smoking status, living place, and other student characteristics are presented in Table 3.

**Table 2:** Association between depression and baseline demographic characteristics.

		Cases and non-cases				Chi-value	*P*-value
		Normal (103)		Depressed (36)			
		*N*	%	*N*	%		
Gender	Male	24	72.7	9	27.3	0.043	0.837
	Female	79	74.5	27	25.5		

Age	19	12	80.0	3	20.0	3.545	0.315
	20	19	61.3	12	38.7		
	21	56	76.7	17	23.3		
	22+	16	80.0	4	20.0		

Nationality	Jordanian	95	73.1	35	26.9	1.097	0.295
	Other nationalities	8	88.9	1	11.1		

Birth order	1	31	83.8	6	16.2	2.419	0.659
	2	13	76.5	4	23.5		
	3	13	68.4	6	31.6		
	4	9	69.2	4	30.8		
	5+	20	71.4	8	28.6		

Year of study	Year 1	18	81.8	4	18.2	6.972	0.073
	Year 2	14	53.8	12	46.2		
	Year 3	35	77.8	10	22.2		
	Year 4	36	78.3	10	21.7		

Father education	Secondary school or below	44	67.7	21	32.3	2.549	0.110
	Above secondary school	52	80.0	13	20.0		

Mother education	Secondary school or below	54	69.2	24	30.8	2.151	0.143
	Above secondary school	42	80.8	10	19.2		

**Table 3:** Association between depression and other student characteristics.

		Cases and non-cases				Chi-value	*P*-value
		Normal		Depressed			
		*N*	%	*N*	%		
Time of decision to be a nursing student	Before high school	16	69.6	7	30.4	0.355	0.837
	During the high school	30	75.0	10	25.0		
	After high school	44	75.9	14	24.1		
Family history of depression or other psychiatric disorders	Yes	7	77.8	2	22.2	0.058	0.810
	No	86	74.1	30	25.9		
Academic performance-any drop in evaluation grades	Yes	21	58.3	15	41.7	6.597	0.010*
	No	64	81.0	15	19.0		
Smoking status	Yes	9	56.2	7	43.8	3.089	0.079
	No	83	76.9	25	23.1		
Place of living	Family	78	71.6	31	28.4	1.541	0.215
	Friends or students hostel	13	86.7	2	13.3		

There was a significant difference in the prevalence of depression between students whose academic performance and grades were deteriorating (41.7%) and those whose grades were stable (19%) (*P* = 0.01).

### Career Choice and Its Association with Depression

Table 4 describes the relationship between depression status and factors affecting career choice. Students who had a desire to help and care for others were less likely to suffer from depression than those who did not, with 22.0 and 40.9%, respectively (*P* = 0.065). Students who chose nursing as their career due to religious factors were significantly less depressed than those who did not, with 19.1 and 40.9%, respectively (*P* = 0.007). Students who chose nursing as their career because of family decisions were significantly more depressed than those who did not, with 31.4 and 13.5%, respectively (*P* = 0.038). Students who chose nursing because there were no other opportunities were significantly more depressed than those who did not, with 47.6 and 18.2%, respectively (*P* = 0.001).

**Table 4:** Association between depression and career choice factors.

Career choice factors		Cases and non-cases				Chi-value	*P*-value
		Normal		Depressed			
		*N*	%	*N*	%		
Past experience with a loved one or self being ill and/or hospitalized	Yes	15	68.2	7	31.8	0.582	0.446
	No	76	76.0	24	24.0		
Your self being ill and/or hospitalized	Yes	14	73.7	5	26.3	0.001	0.974
	No	77	74.0	27	26.0		
Desire to help and care for others	Yes	78	78.0	22	22.0	3.402	0.065
	No	13	59.1	9	40.9		
Religious factors	Yes	76	80.9	18	19.1	7.344	0.007*
	No	13	54.2	11	45.8		
Family decision	Yes	59	68.6	27	31.4	4.298	0.038*
	No	32	86.5	5	13.5		
Family member or friend who was a nurse	Yes	55	72.4	21	27.6	0.434	0.51
	No	35	77.8	10	22.2		
Television/media influence	Yes	56	74.7	19	25.3	0.026	0.872
	No	33	73.3	12	26.7		
Job security	Yes	60	78.9	16	21.1	3.646	0.056
	No	27	62.8	16	37.2		
Financial problems	Yes	40	69.0	18	31.0	1.095	0.295
	No	48	77.4	14	22.6		
No other opportunities	Yes	22	52.4	20	47.6	10.667	0.001*
	No	54	81.8	12	18.2		

*Alpha level ≤0.05.

Students ranked the three most important factors influencing their career selection as: family decision, religious factors, and desire to help and care for others, with a prevalence of 19.9, 15.7, and 14.2%, respectively.

Nearly half of students (44.0%) decided to be nurses after high school, with no difference between males and females but there was a significant difference among those who decided during high school (females 27.0% and males 3.0%; *P* = 0.036). Job security and financial problems were significantly associated with those who decided to study nursing after high school (*P* = 0.001).

### Depression and Sadness Level

Table 5 shows the relationship between depression status and the level of sadness on a scale of one to seven (1 = “Not at all sad” to 7 = “Very sad”). There was a significant difference between the median level of sadness between depressed and normal students (*P* < 0.005), whereas there was no significant difference between the median of the depth of religious belief (“None whatsoever” to “very strong”) between the two groups (*P* = 0.84).

**Table 5:** Mann–Whitney test of differences between normal and depressed groups in sadness level and religious belief.

		Cases and non-cases		*U*-value	*P*-value
		Normal	Depressed		
How sad do you feel right now?	*N*	99	35	728	≤0.01*
	Mean rank	57.35	96.2		
	Median	1	5		
What is the depth of your religious belief?	*N*	99	35	1,694	0.840
	Mean rank	5	5		
	Median	1.515	1.581		

*Alpha level ≤0.05.

### Factors Associated With Depression among Nursing Students

The results show that religious factors, family decision, and no other career/employment opportunities were independently associated with depression, with the following odds ratio (OR) 0.24, *P* = 0.011; OR 4.92, *P* = 0.012; and OR 3.62, *P* = 0.008, respectively. Students who were not classified as depressed (BDI-II score < 17) were 76% more likely to have chosen nursing due to religious factors. However, students classified with depression (BDI-II score ≥ 17) were 4.92 and 3.62 times more likely to have pursued a nursing career due to family pressures and nursing as the only available undergraduate study opportunity, respectively.

## Discussion

The findings in this study indicate that there is a high prevalence of depression among Jordanian nursing students and that depressive symptoms may be related to family pressure in choosing a nursing career. Previous research with nursing students has only explored the prevalence of depression and its association with socio-demographic, coping, and performance factors (17, 20). Some studies focused on the motivating factors affecting their choices to be a nurse (28, 35). Our study is the first to explore the relationship between religiosity and depression.

More than one-quarter (26%) of nursing students in our study were classified with depression and this is higher than the prevalence reported in Jordanian undergraduate students in other professions (34). The prevalence reported in our study was considerably higher than prevalence reported in other studies that investigated nurses: Thessaloniki (Greece) 2006–2007 18% and 14% moderate-to-severe depression (BDI-II score ≥ 20) for 2006–2007 (18) and 2008–2009 (19, 20), and Shiraz (Iran) 10% moderate-to-severe depression (BDI-II score ≥ 20) (22). Possible reasons for the differences in the prevalence of depression include slightly modified cutoff values, different translated versions of the BDI-II, and life-time prevalence of depression rather than point prevalence.

Significant factors affecting career choice were more or less consistent with other studies [family decision, religious factors, and desire to help and care for others (35)]. Furthermore, Kersten and colleagues (28) reported that nurturance, emotional needs, employment opportunities, financial benefits, and interest in science were more prevalent motivating factors in a student’s choice for a career in nursing.

We found that depression status was either negatively or positively associated with nursing career choice factors. The religious factor may be a protective factor as students who were more religious were less likely to be depressed than those who were not; however, future studies using longitudinal designs will need to confirm the temporality of this relationship. Meanwhile, family pressure and a lack of other opportunities for alternative career paths were positively associated with depression among nursing students.

In our study, the majority of the students decided to pursue a nursing career after high school which is in contrast to a previous study that reported students deciding on their future nursing careers during high school (35). Timing of the decision to pursue a career in nursing may be an important factor in the level of depressive symptoms along with the other factors that influence the student’s decision. We found that the time of decision was not statistically significant with respect to depressive symptoms. Interestingly, the time of decision was significantly associated with job security and financial problems. Hamid and colleagues (38) also reported financial problems as one of the factors strongly associated with depressive symptoms among Jordanians women. Moreover, Nasir and Al-Qutob (39) reported that poverty was positively associated with depression in the Jordanian community.

Two earlier studies reported that depressive symptoms tend to increase among students in their second and third years (19, 20). This may be due to students approaching the transition from academic to clinical life (20); however, our study did not find any significant differences in depressive symptoms between years of study. Academic performance was found to be lower among the group of students with depressive symptoms in our study. This result is supported by earlier research that reported a relationship between a high level of academic impairment and the severity of depression (40). Distress among nursing students with poor adjustment and coping skills can result in significant psychiatric morbidity and may interfere with academic performance and even cause withdrawal from the course of study (7).

Although we found a negative relationship between students that decided to pursue a nursing career for religious reasons and depression, there was no association between self-perceived depth of religious belief and classification of depression (normal mood vs. depressed). This observation is inconsistent with other work (41, 42). For example, Papazisis and colleagues (42) found that religious and/or spiritual beliefs among nursing students in Cyprus positively influenced self-esteem and had negative correlation with depression, current stress, and stress as personality trait. Future studies may want to explore the role of different religions on depression in nursing students.

### Limitations

The main limitation of the study is that a cross-sectional study design does not permit determination of the temporality or causality of the relationship between the various factors and depression. Although we recruited a large random representative sample of nursing students, our findings are only generalizable to nursing students at JUST and not all nursing students in Jordan.

### Future Implication

The study findings highlight the importance of developing and implementing strategies and programs to monitor and improve psychosocial health and well-being among nursing students. In addition, students should be provided with effective career counseling to empower them to make the right career decisions for their short- and long-term mental health.

## Conclusion

Our study revealed that more than a quarter of Jordanian nursing students were classified with depression and the main factors associated with depression were family pressure to pursue a nursing career and having no other career or employment opportunities. On the contrary, religiosity was negatively associated with depression and may act as a protective factor; however, future studies using longitudinal designs will need to confirm this hypothesis. High school life and the transitional period before university are critical in framing a student’s psychological well-being. The majority of students who participated in this study decided to be a nurse after high school; therefore, future studies may want to consider using longitudinal designs to track depression and its relationship with career choice from late-adolescence into adulthood.

## Ethics Statement

This study was carried out in accordance with the recommendations of the Institutional Review Board (IRB) of King Abdullah University Hospital, JUST/Jordan. All subjects gave written informed consent in accordance with the Declaration of Helsinki. The protocol was approved by the IRB of King Abdullah University Hospital, JUST/Jordan (No. 3/2012-48).

## Author Contributions

SY conceived the idea, MA and HA collected data, EM analyzed data and HM prepared the manuscripts, IE, TL, and FA-M interpreted results, and all authors reviewed the manuscript.

## Conflict of Interest Statement

The authors declare that the research was conducted in the absence of any commercial or financial relationships that could be construed as a potential conflict of interest.
