# Supplementary material for: Association between Depression and Factors Affecting Career Choice among Jordanian Nursing Students
Source: Front Public Health. 2017 Nov 22;5:311. doi: 10.3389/fpubh.2017.00311 (PMC5703015; doi:10.3389/fpubh.2017.00311)
Supplement: Supplementary file 1 [file data_sheet_1.pdf]

Factors influencing career choice of nursing students

1. Nationality: ……………………………..
2. Birth order in the family…………………
3. Time of the decision to be a nursing student:
   □ Before high school □ During the high school □ After high school
4. Do you have any family history of depression or other psychiatric disorders?

   □Yes □ No

   If yes specify ……………………………………………..

5. Have you been treated or diagnosed as suffering from any psychiatric disorder?
   □Yes □ No
   If yes specify ……………………………………………..
6. Academic performance- any drop in evaluation grades?
   □ Yes □ No
7. Smoking?
   □ Yes □ No
8. Living with
   □ Family □ Friends (student hostel)

	**Factors**	**Yes**	**No**
1	Past experience with a loved one or self being ill and/or hospitalized<br>**If yes**- who is this person………………………		
2	Your self being ill and/or hospitalized<br>**If yes** - how long hospitalized…………………..		
3	Desire to help and care for others		
4	Religious factors		
5	Family decision		
6	Family member or friend who was a nurse		
7	Television/media influence		
8	Job security		
9	Financial problems		
10	No other opportunities		
11	Other (Specify<br>…………………………………………………………………………<br>………………………………………………………………………...		

Please would you rank the first 3 factors according to their importance to you (writ the numbers only): 1-……………….……2-…………………….……..3-…………………..………..
