# Supplementary material for: Association between Depression and Factors Affecting Career Choice among Jordanian Nursing Students
Source: Front Public Health. 2017 Nov 22;5:311. doi: 10.3389/fpubh.2017.00311 (PMC5703015; doi:10.3389/fpubh.2017.00311)
Supplement: Supplementary file 2 [file data_sheet_2.pdf]

BECK DEPRESSION INVENTORY (ARABIC)

تقييم بك للاكتئاب

يتكون هذا الإستبيان من 21 مقولة. نرجو قراءة كل مجموعة بدقة ومن ثم اختيار الجملة التي تناسبك في كل مجموعة والتي تصف شعورك خلال الاسبوعين الماضيين إلى اليوم. ضع دائرة حول الرقم المناسب الذي اخترته. إذا كانت هناك أكثر من إجابة تناسب شعورك ضع دائرة حول الاجابة التي تحمل الرقم الأعلى. نرجو التأكد من عدم وضع دائرة على أكثر من إجابة وذلك يتضمن المجموعة 16 (الشهية) و (النوم)18

**8) Self-Criticalness** **8-إنتقاد الذات**<br>0 .لا أنتقد نفسي ولاألومها أكثر من المعتاد<br>1 أنتقد نفسي لأي ضعف أو خطا أكثر من المعتاد<br>2 أنتقد والوم نفسي على كل أخطائي<br>3 الوم نفسي على كل شيء رديء قد يحصل	**(1) Sadness** 1-**الحزن**<br>0 لا اشعر بالحزن<br>1 اشعر بالحزن أكثر الأحيان<br>2 أشعر بالحزن كل الأوقات<br>3 أنا حزين جدا لدرجة عدم التحمل
**(9) Suicidal Thoughts/Wishes** 9-**تمني الانتحار**<br>0- لا تراودني أي أفكار بقتل نفسي<br>1 - تراودني أفكارا بقتل نفسي ولكن لا أقوم بتنفيذها<br>2-أتمنى لو أقوم بقتل نفسي<br>3-أتمنى أن أقتل نفسي لو تسنح لي الفرصة	**(2) Pessimism** **2-التشاؤم**<br>0 أنا متشجع ومتفائل نحو مستقبلي<br>1 أنا غير متفائل نحو مستقبلي كما في السابق<br>2 أشعر أن ليس لدي ما أتطلع اليه<br>3 أشعر أن مستقبلي بلا أمل وسيزيد سوءا
**(10) Crying** 10-**البكاء**<br>0 لا ابكي أكثر من المعتاد<br>1 .أبكي أكثر من المعتاد<br>2 .ابكي على أقل الأشياء<br>3 .أحس بالرغبة في البكاء ولكني لا أستطيع	**(3) Past Failure** **3-الفشل السابق**<br>0 لا أشعر بالفشل<br>1 أشعر أني فشلت أكثر من الشخص العادي<br>2 حين أنظر إلى الوراء أرى أني فشلت كثيرا<br>3 أشعر أنني شخص فاشل
**(11) Agitation** 11-**الإثارة**<br>0 لا أشعر بالإثارة والتوتر أكثر من المعتاد.<br>1 أشعر بالإثارة والتوتر أكثر من المعتاد<br>2 أشعر بالتوتر والاثارة لدرجة أني لا أستطيع الجلوس<br>3 أشعر بالإثارة والتوتر لدرجة أني استمر بالتحرك أو القيام بأي عمل	**(4) Loss of Pleasure** 4- **فقدان الاحساس بالمتعة**<br>0 استمتع بالأشياء التي أقوم بها كما في السابق<br>1 لا أستمتع بالأشياء التي أقوم بها كما في السابق<br>2 أحصل على متعة قليلة من الأشياء التي كنت أستمتع بها<br>3 لا أشعر بأي متعة نحو أي عمل
**(12) Loss of Interest** 12- **عدم الاهتمام**<br>0 .لم أفقد الاهتمام بالآخرين ولا بالأعمال التي أقوم بها<br>1 .أنا اقل إهتماما بالآخرين وبالأشياء التي أقوم بها<br>2 .اشعر أني فقدت الإهتمام بالآخرين وبالأشياء التي أقوم بها<br>3 أشعر حاليا أنه من الصعب أن اشعر بالإهتمام لأي شيء	**5) Guilt Feelings** **5-الاحساس بالذنب**<br>0 لا اشعر بالذنب بصورة خاصة<br>1 أشعر بالذنب نحو بعض الأشياء التي قمت بعملها او لم أقم بعملها<br>2 أشعر بالذنب معظم الوقت<br>3 أشعر بالذنب طوال الوقت
**(13) Indecisiveness** 13- **عدم القدرة على أخذ القرار**<br>0 أقوم باتخاذ القرارات بطريقتي المعتادة وبنفس الجودة<br>1 أؤجل إتخاذ القرارت أكثر من المعتاد<br>2 أجد صعوبة في إتخاذ القرارات أكثر من المعتاد<br>3 لا أستطيع اتخاذ القرارات	**(6) Punishment Feelings** **6- الاحساس بالعقاب**<br>0 لا أشعر أني أعاقب<br>1 أشعر أنني ربما أعاقب<br>2 أتوقع أن اعاقب<br>3 اشعر أني أعاقب في الوقت الحاضر
**(14) Worthlessness** 14- **الشعور بعدم القيمة**<br>0 لا أشعر بأني بلا قيمة<br>1 اجد أني ذات قيمة كما كنت في السابق<br>2 أشعر أنني بلا قيمة مقارنة بالآخرين<br>3 أشعر أنه ليس لدي أي قيمة	**(7) Self-Dislike** **7- عدم حب النفس**<br>0 لا أشعر بخيبة أمل من نفسي<br>1 اشعر أني فقدت الثقة بنفسي<br>2 أشعر بالخيبة من نفسي<br>3 لا أحب نفسي

**18) Changes in** Appetite **18-تغير بالشهية**<br>0 لم أشعر بأي تغير بشهيتي<br>1 اشعر أن شهيتي أقل من الطبيعي<br>2 أشعر أن شهيتي أكثر من الطبيعي<br>3 أشعر أن شهيتي خفت عن قبل<br>4 أشعر أن شهيتي زادت عن قبل<br>5 أشعر أنه ليس لدي شهية أبدا<br>6 أشعر بالرغبة في الطعام دائما	**(15) Loss** of Energy **15- فقدان الطاقة**<br>0 لدي الطاقة وبامكاني العمل تقريبا بنفس الجودة<br>1 أشعر أن معدل الطاقة لدي أقل مما كان عليه<br>2 ليس لدي طاقة كافية للقيام بالكثير من الأعمال<br>3 ليس لدي طاقة لأقوم بأي شيء
**19) Concentration Difficulty** 19- **صعوبة التركيز**<br>0 أستطيع التركيز كما في السابق<br>1 لا أستطيع التركيز كما في السابق<br>2 لا استطيع أن اركز تفكيري على شيء واحد لفترة طويلة<br>3 اجد أنني لا أستطيع التركيز على أي شيء	**(16) Changes in Sleeping Pattern 16- تغير بعادات النوم**<br>0 لم تتغير عادات نومي<br>1 انام اكثر من الطبيعي<br>2 انام أقل من الطبيعي<br>3 أنام أكثر بكثير من الطبيعي<br>4 انام اقل بكثير من الطبيعي<br>5 انام أكثر اليوم<br>6 أصحو من النوم ابكر بساعة إلى ساعتين ولا أستطيع النوم بعدها
**(20) Tiredness or Fatigue 20-التعب والارهاق**<br>0 لا أشعر بالإرهاق أكثر من المعتاد<br>1 أشعر بالإرهاق والتعب أكثر من المعتاد<br>2 .أشعر أنني متعب ومجهد ولا أستطيع القيام بأكثر الأشياء التي كنت أقوم ممارستها<br>3 أشعر أنني متعب ومجهد دائما ولا أستطيع القيام بكل الأشياء التي كنت أمارسها من قبل	**(17) Irritability 17-سهولة الانزعاج**<br>0 لا انزعج بسرعة أكثر من المعتاد<br>1 أشعر بالإنزعاج أكثر من المعتاد<br>2 هذه الأيام أشعر بالإنزعاج كثيرا<br>3 أشعر بالإنزعاج دائما
	**(21) Loss of Interest in Sex 21-فقدان الرغبة بالجنس**<br>0 لم الاحظ أي تغيرات جديدة في رغبتي الجنسية<br>1 رغبتي الجنسية أقل مما كانت عليه<br>2 حاليا أنا أقل بكثير اهتماما بالجنس<br>3 لقد فقدت رغبتي الجنسية تماما
